# Genome Sequence of Lysinibacillus sphaericus, a Lignin-Degrading Bacterium Isolated from Municipal Solid Waste Soil

**DOI:** 10.1128/genomeA.00353-18

**Published:** 2018-05-03

**Authors:** Gabriela F. Persinoti, Douglas A. A. Paixão, Timothy D. H. Bugg, Fabio M. Squina

**Affiliations:** a Laboratório Nacional de Ciência e Tecnologia do Bioetanol (CTBE), Centro Nacional de Pesquisa em Energia e Materiais (CNPEM), Campinas, São Paulo, Brazil; b Department of Chemistry, University of Warwick, Coventry, United Kingdom; c Programa de Processo Tecnológicos e Ambientais da Universidade de Sorocaba (UNISO), Sorocaba, Brazil

## Abstract

We report here the draft genome sequence of Lysinibacillus sphaericus strain A1, a potential lignin-degrading bacterium isolated from municipal solid waste (MSW) soil and capable of enhancing gas release from lignocellulose-containing soil.

## GENOME ANNOUNCEMENT

Lysinibacillus sphaericus strain A1 is a Gram-positive bacterium from the *Firmicutes* phylum that was isolated from municipal solid waste (MSW) soil in the United Kingdom (1). This lignin-degrading bacterium strain is particularly interesting since, under microscale anaerobic conditions, it presents enhanced methane release from lignocellulose-containing soil, suggesting the potential for *in situ* treatment and enhancement of landfill soil gas production (1).

We report here the genome sequence of *L. sphaericus* strain A1. We sequenced the genome of this strain on an Illumina HiSeq 2500 system at the CTBE next-generation sequencing (NGS) facility, generating 5,262,224 paired-end reads (insert size, 300 bp) and 4,454,143 mate pair reads (3 libraries with an insert size between 3 and 4 kb, 5 and 7 kb, and 8 and 11 kb). Paired-end reads were preprocessed with Trimmomatic (2) to remove the adapter and low-quality sequences, and mate pair reads were processed using NextClip (3), resulting in 5,178,788 and 1,227,816 high-quality reads, respectively.

The genome size of *L. sphaericus* was estimated to be 4.54 Mb based on k-mer count statistics accessed with KmerGenie (4), with an estimated coverage of approximately 200×. Genome assembly was carried out with SPAdes v3.6.2 (5) using an assembly of k-mer values (21, 33, 47, 55, and 77) and SSPACE (6). The presence of typical bacterial marker genes in the assembled genome was assessed using CheckM (7), which estimated the completeness of the genome to be 99.91%.

The resulting assembly for *L. sphaericus* has nine scaffolds with a total length of 4,517,188 bp and an *N*_50_ value of 3,305,581 bp. The average GC content of the genome is 37%.

Gene prediction and annotation were carried out using the Prokka prokaryotic genome annotation pipeline (8). A total of 4,371 genes were identified in the *L. sphaericus* genome; of these, there are 4,305 protein-encoding genes, 9 rRNAs, 56 tRNAs, and 1 transfer-messenger RNA (tmRNA). Regarding 16S rRNA, *L. sphaericus* strain A1 presents 99.92% sequence similarity with *L. sphaericus* KCTC 3346^T^, 99.76% with L. fusiformis NBRC 15717^T^, and 99.60% with L. mangiferihumi M-GX18^T^, as analyzed using the EzBioCloud Web server identity tool (9).

### Accession number(s).

This whole-genome shotgun project has been deposited at DDBJ/ENA/GenBank under the accession number PGLV00000000. The version described in this paper is version PGLV01000000.
